# The Utility of Ovotransferrin and Ovotransferrin-Derived Peptides as Possible Candidates in the Clinical Treatment of Cardiovascular Diseases

**DOI:** 10.1155/2017/6504518

**Published:** 2017-03-13

**Authors:** Shuang Chen, Hongmei Jiang, Hanhui Peng, Xiaosong Wu, Jun Fang

**Affiliations:** ^1^College of Bioscience and Biotechnology and College of Animal Science and Technology, Hunan Agricultural University, Changsha 410128, China; ^2^Hunan Province University Key Laboratory for Agricultural Biochemistry and Biotransformation, Hunan Agricultural University, Changsha 410128, China; ^3^Hunan Co-Innovation Center for Utilization of Botanical Functional Ingredients, Changsha 410128, China

## Abstract

Several of the most prevalent etiological factors which contribute towards global death rates are associated with cardiovascular diseases (CVDs), which include a range of conditions such as angina, rheumatic heart disease, and venous thrombosis. Extensive research has been conducted into the role played by oxidative stress and inflammation in the functional transformations associated with the progression of CVDs, while the research findings from these investigations have been both fruitful and informative. In view of the adverse secondary effects that result from the clinical administration of many synthetic medications, research which explored the treatment of severe and long-lasting conditions, including CVDs, has primarily centered on the potential benefits displayed by natural agents, one of which is food protein-based bioactive peptides. Most importantly, previous research has revealed the possible benefits associated with these products' anti-inflammatory and antioxidant characteristics. In light of these considerations, this paper aims to review the degree to which ovotransferrin (otrf, also referred to as conalbumin) and otrf-derived peptides, including IRW, IQW, and KVREGT, are, by virtue of their anti-inflammatory and antioxidant characteristics, viable treatment agents for endothelial dysfunction and the prevention of CVD.

## 1. Introduction

As has previously been determined by the analysis of statistical evidence, cardiovascular diseases (CVDs), which include conditions such as angina, rheumatic heart disease, and stroke, account for an alarming proportion of the global annual death rate [1, 2]. Statistics from 2013 indicate that two types of CVD, myocardial infarction and stroke, resulted in 249.7 fatalities per 100,000 individuals, thereby causing 28.2% of the deaths globally that year [3]. Previous research has evidently shown that the most prevalent CVDs are cerebrovascular disease and coronary artery disease, while these conditions are emerging as a consequence of the gradual progression of lesions in the arteries in conjunction with luminal narrowing; specifically, this is referred to as atherosclerosis. The lesions associated with atherosclerosis are constituted by several immune response cells, including T-cells, in combination with cholesterol; moreover, the rise in the number of cases of CVDs as a category has been linked to the lifestyle-based transformations that began to occur from the 1950s in the developed world, primarily due to the proliferation of industrialisation, urbanised living, and advancing economies [4–6]. Although modifiable and controllable risk factors play a prominent role in determining the onset of CVDs, including hypertension, high blood sugar levels, smoking, and lack of exercise, a range of factors remain unchanged [7]. The most notable risk factors in the latter category are demographic by nature, and they include an individual's age, gender, and genetic constitution.

One of the critical aspects that must be acknowledged when seeking to treat, prevent, and manage atherosclerosis and CVDs is that many of the available pharmaceutical agents result in adverse secondary effects [8]. As a direct response to this, researchers have sought to mitigate these negative impacts by identifying nonsynthetic (namely, natural) alternatives, many of which are not associated with major secondary effects. Naturally occurring proteins and the peptides that comprise them have been identified as candidates of considerable promise in this respect, primarily owing to the innovative ways in which compounds with beneficial biological impacts can be formulated from them [9, 10]. Ovotransferrin (otrf, also referred to as albumin), a glycoprotein associated with egg white albumen belonging to a category of transferrin iron-binding glycoproteins, is one such naturally occurring protein. Otrf constitutes approximately 12-13% of egg albumen; owing to the capacity it has to impede the progression of microorganisms, it is a key component in determining the success of the developmental process for chicken embryos [11]. However, otrf was not only able to impede the growth of bacteria through iron holding; recent research has discovered a range of additional beneficial factors which positively contribute towards the advancements of developing embryos. To be specific, these include a regulatory role in the absorption of Fe^3+^, antiviral (along with antibacterial and anti-inflammatory) characteristics, and immune response.

Therefore, the purpose of the present paper is to evaluate the viability of otrf and otrf-derived peptides, owing to the capacity they have to modulate oxidative stress and inflammation, as candidates for the maintenance of endothelial operations and the prevention of endothelial dysfunction.

## 2. Impacts of Oxidative Stress and Inflammation on Endothelial Function in CVDs

Recently conducted research has focused on the role played by the endothelium in the context of vascular physiology; as a result of this research, knowledge of the subject has considerably increased. The present state of anatomical knowledge on the subject indicates that the vascular endothelium is the human body's most expansive endocrine organ, while Zachary reports that the secretion of a range of vasoactive agents takes place by way of endothelial cells [12]. As was noted in the paper, the vasoactive agents which have so far been identified include nitric oxide, prostacyclin, and the endothelium-derived hyperpolarising factor (vasodilatory agents), along with endothelin I, angiotensin II, and thromboxane (vasoconstrictor agents). As a vasodilatory agent, nitric oxide facilitates the relaxation of blood vessels while it is also linked to the prevention of thrombus formation, the suppression of smooth muscle proliferation, and the impedance of the attachment of leukocytes to the activated endothelium [13]. Findings from the last two decades have indicated a range of critical functions associated with the endothelium, including regulating vascular tone, inhibiting vascular growth and platelet coagulation, modulating inflammation, and contributing towards the onset of atherosclerosis [13]. Moreover, as identified by Albarran et al., the pathophysiology of metabolic syndrome and a range of CVDs are associated with endothelial dysfunction [14]. With regard to endothelial dysfunction, it has also been determined that the diminished bioavailability of endothelium-derived nitric oxide plays a critical role. To be more specific, Münzel et al. stated that a uniform feature associated with every cardiovascular risk factor is the reduction in endothelial nitric oxide bioavailability; as a result of this, it is possible to predict the condition's advancement as well as determine the outcome [15]. It should also be acknowledged that several processes associated with the pathology of CVDs play an intermediary role in endothelial dysfunction, and this relates to overactivity regarding the renin-angiotensin system (RAS), oxidative stress, and dysregulated inflammation.

In cases where the rate at which oxidants form surpasses the ability of the body's antioxidant prevention mechanisms, oxidative stress is said to have taken place. The creation of reactive oxygen species (ROS) is related to an increase in blood pressure. ROS facilitates this increase in blood pressure in two main ways: firstly, it facilitates hypertrophy, media thickening, and collagen deposition, thereby resulting in vascular remodeling [16]; secondly, it inactivates nitric oxide, thereby promoting endothelial dysfunction [16, 17]. The latter consequence results from the fact that nitric oxide serves as the main vasodilatory agent in the endothelium [16, 17]. In cases where a patient is obese due to dietary factors, oxidative stress facilitates a range of signaling pathways which have a high degree of sensitivity to stress. Subsequently, this leads to the phosphorylation of important proteins in insulin signaling, the chief implication of which is to inhibit the translocation regarding various subcellular compartments [18]. This is one of the key etiological factors underlying the development of type 2 diabetes mellitus (T2DM), primarily due to the fact that it facilitates resistance to insulin [18]. Another effect of ROS is the activation of leukocyte-derived proteases; these facilitate arterial remodeling through the inducement of the vascular wall and interstitial matrix proteolysis [19, 20].

The excessive generation of ROS stems from the way in which vascular inflammation leads to the emergence of a range of cytokines. In view of this, research has discovered that this lowers nitric oxide bioavailability while a decrease in nitric oxide bioavailability results in endothelial dysfunction while constituting a significant risk factor for hypertension [21]. This finding is corroborated by Panagiotakos et al. who studied a sample of individuals suffering from high blood pressure whereby they reported a heightened rate of inflammatory factors in cardiovascular control organs [22]. It is important to recognise that the overexpression of inflammatory factors has the potential to result in the formation of vascular plaque; when combined with oxidised lipid molecules, the primary consequence is the onset of vascular remodeling and hypertension [23]. Kohlgruber and Lynch noted that cellular insulin resistance is facilitated by adipose tissue macrophages (ATM) due to the fact that they promote the secretion of a range of cytokines in a way that is directly proportional to an individual's level of obesity; these cytokines lead to the activation of kinases which subsequently result in the phosphorylation of insulin signaling molecules [24]. In addition to macrophages, past research identified lymphocytes in adipose tissues as etiological factors in inflammation and insulin resistance [25]. This stems from the way in which they facilitate the accumulation of macrophages in adipose tissues [25].

## 3. Characteristics of Ovotransferrin

One of the key motivations underlying the recent initiatives among researchers to evaluate the potential displayed by naturally occurring proteins as possible candidates for the treatment of CVDs stems from the desire to bypass the adverse secondary effects associated with synthetic pharmaceutical agents. Added to this is the knowledge that any alternative means that can be employed to counteract the high levels of morbidity associated with CVDs in the contemporary world are urgently required.

Ovotransferrin (otrf), a monomeric glycoprotein constituted by 686 amino acids, contains an isoelectric point of 6.0 and a molecular weight of 77.9 kDa that was produced at a high level in the egg white [26]. Otrf is classified as a superoxide dismutase-mimicking protein while research has discovered that it creates superoxide radical (O_2_^−^)-scavenging activity. The liver and the oviduct facilitate the transcription of the avian transferrin gene; in the former, the secretion of transferrin takes place within the serum. Subsequently, it participates in the transferral and storage of Fe^3+^. In regard to the latter, the oviduct, the secretion of transferrin (referred to as ovotransferrin in this particular case) takes place at a high rate in the egg whites.

The broad range of defensive operations afforded by otrf has already been overviewed; from the analysis, it is evident that the critical dimension is its antibacterial capacity. It is important to recognise that its antibacterial capacity is associated with the way in which it can bind Fe^3+^. In a study conducted by Ko et al., the researchers presented otrf, NaHCO_3_, and EDTA as examples to reveal that there is a rise in the bacteriostatic activity regarding* E. coli* O157:H7 as iron chelator [27]. Furthermore, an in vivo research conducted on infant guinea pigs that were subject to* E. coli* (0111 B4) oral infection also revealed otrf's antibacterial characteristics [27, 28]. Additional research has been conducted which suggests that the antibacterial characteristics of otrf are not solely linked to the extrication of iron from the medium; the studies have instead demonstrated that the antibacterial functions are associated with the binding of otrf directly to the surface of bacterial organisms. In another study involving* E. coli* conducted by Aguilera et al. [29], it was evidently proven that otrf has the capacity to penetrate the outer membrane of the bacteria and then subsequently enter the inner membrane. As documented by the researchers, this leads to the ion leakage in combination with the uncoupling of the respiration-dependent energy production. Studies which utilised another type of bacteria,* Salmonella enterica *(serovar* Choleraesuis*), noted that the glycoprotein's antibacterial efficacy relies on culture variables associated with the effectiveness of otrf's binding to the surface of the bacterial organisms [30, 31].

With regard to the antiviral characteristics of otrf, the results suggested that the capacity which the glycoprotein is able to mitigate against Marek's disease virus (MDV) stems from the following otrf fragments: DQKDEYELL and KDLLFK [32, 33]. Despite the fact that blocking efficiency in regard to the infection is less for the isolated peptides than with the intact protein, the studies further indicate that these fragments are effective in blocking MDV in chicken embryo fibroblasts. It is interesting to note that, from an evolutionary perspective, DQKDEYELL and KDLLFK display common sequence homology in comparison to two protein fragments that are generated from human and bovine lactoferrin; moreover, past research has demonstrated that these have a high degree of efficacy when applied to the* Herpes simplex* virus (HSV-1) [34].

A comparative examination of the antioxidant characteristics of hen's otrf (and its hydrolysed peptides) and superoxide anion scavenging activity revealed the former to be around 3.2–13.5 times greater [35]. Moreover, otrf-derived peptides displayed synergistic antioxidant impacts associated with vitamin C, epigallocatechin gallate, and caffeic acid [36]. Otrf hydrolysate displayed defensive capacity regarding oxidative stress associated with DNA lesions in human leukocytes [37]. In addition, Moon et al. uncovered evidence showing otrf's metal-chelating characteristics in association with the antioxidant processes [38].

Rath et al. suggested the utility of otrf concentration in the blood as an indicator of infection and inflammation in avian species [39]. This relates to the findings which show that avian inflammation processes display upregulation of otrf in vivo and in vitro [40]; furthermore, Xie et al. also revealed how blood otrf concentration is increased over the duration of the inflammation process [41].

## 4. IRW, IQW, and KVREGT

Huang et al. noted that a staple feature in the clinical treatment of hypertension is antioxidants, which are primarily phenolic compounds [42]. Research has also revealed that the production of egg tripeptides (namely, IRW and IQW) from otrf takes place, while the efficacy of this has been presented regarding the downregulation in the expression of the cytokine-induced inflammatory protein in the vascular endothelium [43, 44]. The latter two studies suggest that this partially occurs through the modulation of the NF-*κ*B pathway. Researchers posited the anti-inflammatory and antioxidant capacity of IRW on endothelial cells [44], while Majumder et al. corroborated this by employing an in vivo study [45]. Specifically, the study incorporated SHRs to demonstrate that the peptide had antihypertensive properties and, moreover, that it facilitated enhancements to endothelium-dependent vasorelaxation (demonstrated in an ex vivo study). In addition, Majumder et al. found evidence to indicate that enhancements regarding lessened indicators of inflammation, along with significant relationships, were identified with respect to cell-based and animal research [45]. Recent reports testify to the capacity at which IRW has to upregulate ACE2 expression in the context of SHR vasculature [31], while comparable anti-inflammatory properties regarding endothelial cells and vasculoprotective functions have been identified regarding SHRs [46]. Therefore, the capacity at which IRW has to lower blood pressure stems directly from its anti-inflammatory and antioxidative characteristics, combined with the method with which it enhances endothelial function. In each scenario, it is reasonable to suggest that a series of dimensions led to the results: namely, the impedance of ACE, enhanced bioavailability of nitric oxide, and anti-inflammatory capacity. The promise of IQW is also seen regarding the way in which it impedes ACE and facilitates antioxidation [36]; however, it should be acknowledged, as was reported by Majumder et al. [43], that it only has a positive impact when proximate to a viable tripeptide. The researchers have observed how this is due to the isolation of matching dipeptides and constituent amino acids which lack the capacity to produce the anti-inflammatory results. Most importantly, this is indicative of a strong and significant correlation between structure and function regarding the structural features of the tripeptide and anti-inflammatory outcomes [43].

As was noted by Lee et al., the hen otrf's peptide KVREGT revealed an antihypertensive secondary function associated with otrf in that an IC50 value of 9.1 *μ*M was associated with the angiotensin I-converting enzyme [47]. Recent research also revealed that KVREGT performs a significant function in impeding ACE and facilitating vasodilation [48].

## 5. Concluding Remarks

The methods in which CVDs develop have been a primary concern in this study, while the researcher has documented the fact that the critical risk factors associated with the broad range of cardiovascular conditions are understood in the literature. As a result of the negative secondary effects stemming from widely used synthetic treatment agents, the potential factors associated with naturally occurring food-based protein and peptides to serve as alternatives have been rigorously investigated in recent years. In view of the critical role played by oxidative stress and inflammation as underlying etiological factors leading to CVDs, antioxidative and anti-inflammatory peptides show considerable promise as viable candidates. This paper has cited numerous studies which emphasise the defensive capacity of otrf, while it is also notable that several of these are common to its proteolytic fragments, thereby contributing towards the encouragement of the use of egg white as a dietary supplement. However, despite the increasing body of research (primarily cell and in vivo studies) supporting the beneficial impacts of otrf, knowledge of peptide receptors and molecular mechanisms is lacking at present; to be more specific, an understanding of the precise methods with which these operations lead to positive effects regarding hypertension, insulin resistance, and obesity is not yet fully obtained. Therefore, future studies must be conducted in order to increase the scope of our knowledge on these topics, thereby further developing the contemporary body of literature examining the issue of how food-derived peptides can be mobilised in preventing, treating, and managing CVD.
