# Corrigendum to “The Utility of Ovotransferrin and Ovotransferrin-Derived Peptides as Possible Candidates in the Clinical Treatment of Cardiovascular Diseases”

**DOI:** 10.1155/2018/1897257

**Published:** 2018-12-13

**Authors:** Shuang Chen, Hongmei Jiang, Hanhui Peng, Xiaosong Wu, Jun Fang

**Affiliations:** ^1^College of Bioscience and Biotechnology and College of Animal Science and Technology, Hunan Agricultural University, Changsha 410128, China; ^2^Hunan Province University Key Laboratory for Agricultural Biochemistry and Biotransformation, Hunan Agricultural University, Changsha 410128, China; ^3^Hunan Co-Innovation Center for Utilization of Botanical Functional Ingredients, Changsha 410128, China

In the article titled “The Utility of Ovotransferrin and Ovotransferrin-Derived Peptides as Possible Candidates in the Clinical Treatment of Cardiovascular Diseases” [1], there is a missing section that should be added between sections 4 and 5 as follows:

## 5. Research on Ovotransferrin or Ovotransferrin-Derived Peptides in Cardiovascular Disease

Due to the rapid development of biomedicine, many nonconquerable diseases have been alleviated and treated in varying degrees. But inevitably, synthetic drugs have side effects, and there is a strong interest in finding new bioactive substances as potential candidates for treatment. Bioactive peptides exert systemic effects by entering the circulation via the intestinal tract or produce local effects in the gastrointestinal tract. But the most important thing is that it enhances health and safety profiles and may be a candidate for cardiovascular disease treatment [49]. The causes of cardiovascular disease are diverse, such as lifestyle habits, eating habits, and many congenital causes, which plague people's normal life. Studies have shown that ovotransferrin and ovotransferrin-derived peptides could be anti-inflammatory, anti-oxidatory, and inhibit ACE to ease the development of cardiovascular disease (Table 1). Tumor necrosis factor (TNF) is a proinflammatory cytokine, which plays an important role in the development of atherosclerosis. TNF can upregulate the expression of intercellular adhesion molecule-1 (ICAM-1) and vascular cell adhesion molecule-1 (VCAM-1) in endothelial cells, leading to the development and progression of vascular inflammation. Studies have shown that ovalbumin-ovotransferrin peptides (IRW and IQW) could alleviate TNF-induced inflammation and oxidative stress in endothelial cells [43]. RVPSL, a bioactive peptide from egg protein, was found to reduce the blood pressure of spontaneous hypertensive rats and reduce the mRNA expression of renin, ACE, and AT1 receptor in the kidney. In addition, the concentrations of angiotensin II, renin, and aldosterone in serum dropped. This indicated that RVPSL has the potential to treat hypertension [50]. So far, no human-targeted clinical trials of ovotransferrin or ovotransferrin-derived peptides have been carried out, though that does not negate its therapeutic potential. Bioactive peptides from other sources have been studied in hypertensive patients. For example, VPP, an active peptide in fermented milk, has been shown to reduce blood pressure in hypertensive patients [51].

In addition, the following references should have been included in the references list of the original article [1]:

- Erdmann et al. [2] should be added as reference [49].
- Yu et al. [3] should be added as reference [50].
- Seppo et al. [4] should be added as reference [51].
- Liao et al. [5] should be added as reference [52].
- Huang et al. [6] should be added as reference [53].

## Figures and Tables

**Table 1:** The regulators of ovotransferrin and ovotransferrin-derived peptides and adjusting effect.

Bioactive peptide	Regulated factor	The result of adjustment	Reference
IRW	Angiotensin II	Inhibited the migration of vascular smooth muscle cells	[52]
IRW	ACE inhibition and endothelial nitric oxide synthase	Antihypertensive effects	[45]
IRW and IQW	Tumor necrosis factor (TNF)	Reducing endothelial inflammation and oxidative stress	[43]
Ovotransferrin	Reactive oxygen species	Antioxidants and oxygen radical-scavenging effects	[53]
IRW	ACE2, ICAM-1, and VCAM-1	Increased ACE2 and decreased proinflammatory genes expression	[31]
